# Obesity and Weight Gain in Pregnancy and Postpartum: an Evidence Review of Lifestyle Interventions to Inform Maternal and Child Health Policies

**DOI:** 10.3389/fendo.2018.00546

**Published:** 2018-09-26

**Authors:** Nathalie J. Farpour-Lambert, Louisa J. Ells, Begoña Martinez de Tejada, Courtney Scott

**Affiliations:** ^1^Obesity Prevention and Care Program “Contrepoids,” Service of Therapeutic Education for Chronic Diseases, Department of Community Medicine, Primary Care and Emergency, Geneva University Hospitals and University of Geneva, Geneva, Switzerland; ^2^Pediatric Sports Medicine Consultation, Service of General Pediatrics, Department of Child and Adolescent, Geneva University Hospitals and University of Geneva, Geneva, Switzerland; ^3^School of Health and Social Care, Teesside University, Middlesbrough, United Kingdom; ^4^Service of Obstetrics, Department of Gynaecology and Obstetrics, University Hospitals of Geneva and University of Geneva, Geneva, Switzerland; ^5^London School of Hygiene and Tropical Medicine, University of London, London, United Kingdom

**Keywords:** obesity, weight gain, pregnancy, postpartum, physical activity, nutrition, intervention, systematic review

## Abstract

**Background:** Maternal obesity, excessive gestational weight gain (GWG) and post-partum weight retention (PPWR) constitute new public health challenges, due to the association with negative short- and long-term maternal and neonatal outcomes. The aim of this evidence review was to identify effective lifestyle interventions to manage weight and improve maternal and infant outcomes during pregnancy and postpartum.

**Methods:** A review of systematic reviews and meta-analyses investigating the effects of lifestyle interventions on GWG or PPWR was conducted (Jan 2009–2018) via electronic searches in the databases Medline, Pubmed, Web of Science and Cochrane Library using all keywords related to obesity/weight gain/loss, pregnancy or postpartum and lifestyle interventions;15 relevant reviews were selected.

**Results:** In healthy women from all BMI classes, diet and physical activity interventions can decrease: GWG (mean difference −1.8 to −0.7 kg, high to moderate-quality evidence); the risks of GWG above the IOM guidelines (risk ratio [RR] 0.72 to 0.80, high to low-quality evidence); pregnancy-induced hypertension (RR 0.30 to 0.66, low to very low-quality evidence); cesarean section (RR 0.91 to 0.95; high to moderate-quality evidence) and neonatal respiratory distress syndrome (RR 0.56, high-quality evidence); without any maternal/fetal/neonatal adverse effects. In women with overweight/obesity, multi-component interventions can decrease: GWG (−0.91 to −0.63 kg, moderate to very low-quality evidence); pregnancy-induced hypertension (RR 0.30 to 0.66, low-quality evidence); macrosomia (RR 0.85, 0.73 to 1.0, moderate-quality evidence) and neonatal respiratory distress syndrome (RR 0.47, 0.26 to 0.85, moderate-quality evidence). Diet is associated with greater reduction of the risks of GDM, pregnancy-induced hypertension and preterm birth, compared with any other intervention. After delivery, combined diet and physical activity interventions reduce PPWR in women of any BMI (−2.57 to −2.3 kg, very low quality evidence) or with overweight/obesity (−3.6 to −1.22, moderate to very low-quality-evidence), but no other effects were reported.

**Conclusions:** Multi-component approaches including a balanced diet with low glycaemic load and light to moderate intensity physical activity, 30–60 min per day 3–5 days per week, should be recommended from the first trimester of pregnancy and maintained during the postpartum period. This evidence review should help inform recommendations for health care professionals and women of child-bearing age.

## Introduction

Overweight and obesity are increasing steadily in all age groups worldwide, especially in low- and middle income countries (1). Pre-pregnancy obesity (body mass index, BMI ≥ 30 kg/m^2^), excessive gestational weight gain (GWG) and post-partum weight retention (PPWR) are seen as new public health challenges, given the association with negative short- and long-term maternal and child outcomes (2). These outcomes include obstetrical or neonatal complications, obesity, type 2 diabetes (T2D) and cardiovascular diseases (CVD) later in life (3–10) (see Table 1).

**Table 1:** Pre-pregnancy obesity-related risks to women and offspring.

**Period**	**Women**	**Offspring**
Before conception	Menstrual cycle dysregulation, anovulation and infertility (11)	–
Pregnancy	Miscarriage (12) Gestational diabetes mellitus (13) Pregnancy-induced hypertension (14, 15) Preeclampsia (16) Thrombo-embolism (17)	Congenital defects (18, 19) Premature birth (20) Large for gestational age Macrosomia (>4,000 g) (21)
Delivery	Cesarean sections Labor induction, surgical complications and failures of epidural analgesia (3, 22–26)	Stillbirth (24, 27) Neonatal trauma (assisted vaginal delivery and head trauma, shoulder dystocia) (7, 28) Low umbilical arterial pH < 7.1 and low Apgar score at 5 and 10 min (7)
Postpartum	Difficulties in initiating and sustaining breastfeeding (29)	Systematic transfer to monitoring in case of GDM (risk of hypoglycemia) (7) Increased admission rate in the intensive care unit (30)
Long-term	Postpartum weight retention and inter-pregnancy obesity (31, 32) Type 2 diabetes Long-term vascular dysfunction (14, 15)	Childhood obesity and premature metabolic syndrome (33) Premature death from cardiovascular disease (22)

Large for gestational age neonates have a 50% risk of developing obesity and a metabolic syndrome between 6 and 11 years of age (33), and a 35% risk of dying prematurely of CVD (22). To reduce the detrimental intergenerational cycle of obesity and associated non-communicable diseases (NCDs), weight management during pregnancy and postpartum should be prioritized across countries, with an increased commitment for concerted, coordinated and specific actions. The aim of this overview is to draw together systematic review evidence examining the effectiveness of different intervention approaches for the management of maternal weight and the improvement of maternal and child health outcomes.

### Maternal obesity as a global health issue

In 2016, a world report from the Non-Communicable Diseases Risk Factor Collaboration indicated that age-standardized prevalence of obesity increased from 3.2% in 1975 to 10.8% in 2014 in men, and from 6.4 to 14.9% in women (1). In the United States of America (U.S.A), pre-pregnancy obesity prevalence increased by an average of 0.5% point per year from 17.6% in 2003 to 20.5% in 2009 (34). Currently, 31.9% of reproductive age women in the U.S.A. have obesity and 55% have obesity or are overweight, with a higher prevalence in non-Hispanic black and Mexican American women (35). In the European region, the current prevalence of maternal obesity ranges from 7 to 25% (36), and it is expected to increase to 37% by 2020 (22). First trimester maternal obesity is significantly increasing over time in the United Kingdom too, having more than doubled from 7.6 to 15.6% over 19 years (1989 and 2007) (22).

### Gestational weight gain, maternal and neonatal outcomes

Ideally, total GWG is calculated as the difference between body weight at the first trimester and last antenatal visit prior delivery (37). Gestational weight gain differs between individual women (38), and is associated with several factors such as pre-pregnancy BMI, maternal age, parity, ethnicity, GDM, hypertension, edema and smoking (39). Both over- or under-nutrition during gestation, particularly during the first two trimesters, are related to childhood obesity (33, 40, 41). Gestational weight gain is closely associated with the infant's birth weight and every additional kilogram of GWG can increase birth weight by 7.35g (42). Excessive GWG is related to overweight in early, middle and late childhood (43), and later life (40 years) in daughters (44).

Excessive GWG is a known risk factor for multiple adverse outcomes such as GDM, pregnancy-induced hypertension, preeclampsia, stillbirth, macrosomia, and post-partum hemorrhage (45, 46). It also contributes to long-term PPWR in childbearing women, and related disease outcomes, thus elevating the risks for subsequent pregnancies (47, 48). Pre-pregnancy BMI is a strong predictor of excessive GWG (49–51). Baseline overweight combined with excessive GWG results in an increased risk of fetal complications, and a higher long-term likelihood of retaining excessive weight (50, 51). In women with low socio-economic status, high early pregnancy BMI, nulliparity, and discordant clinician advice are directly associated with excessive GWG (52). A meta-analysis of 17 observational studies showed a significant relationship between excessive GWG and higher PPWR risk (OR 2.08; 95% CI: 1.60 −2.70) (53), however mean PPWR decreased with increasing BMI classes. Authors suggested that GWG, rather than pre-pregnancy BMI, determines the shorter or longer PPWR.

Inter-pregnancy weight gain, which may be due to PPWR or additional weight gain between gestations, is also associated with adverse pregnancy outcomes (54), and long-term obesity, type 2 diabetes and risk factors for CVD (55). The average PPWR ranges from 0.5 kg (56) to 4 kg (57); however, 14–25% of women who gain significant amounts of weight during pregnancy will retain more than 4.5 kg after birth (55, 57). Woman in child-bearing years (25–34 years) have the highest risk of weight gain compared with men or women in other age groups (58). In Sweden, two-thirds of women weigh more than their pre-pregnancy weight at 6 months postpartum (59) and, in the U.S.A., up to 75% of low-income postpartum mothers are heavier at 1 year postpartum compared with their pre-pregnancy weight (60). In a longitudinal study of 2055 postpartum women in Australia, a greater postnatal increase in BMI was reported for women defined as having excessive GWG (odds ratio 3.72; 95% CI: 3.12–4.31) than for women with adequate GWG. Those who gained excess weight during pregnancy had increased odds of being overweight (2.15; 95% CI: 1.64–2.82) or to have obesity (4.49; 95% CI; 3.42–5.89) 21 years after the index pregnancy (49). So, failure to lose excessive GWG after delivery can contribute to obesity in midlife, and to an intergenerational cycle of obesity within the female population and offspring.

### Current maternal weight policies

Despite the growing evidence that maternal obesity and excessive GWG are risk factors for major obstetrical complications, poor subsequent maternal and child health, and for the transmission of obesity to the next generations (61, 62), there is inconsistency in maternal weight gain policies across the world (63). This may be explained by the fact that there is some evidence that women with obesity may have better outcomes if they gain only small amounts of weight, or even lose weight during pregnancy, while there is conflicting evidence that insufficient GWG may result in increased risk of intra-uterine growth retardation (IGR) and small for gestational age (SGA) (64–68). Coherence in guidelines internationally is important to address both inadequate and excessive GWG, including all obesity classes.

In 1990, the U.S.A. IOM produced guidelines for GWG which were been updated in 2009 (Table 2) (69). These guidelines are widely considered to be the international gold standard, however they do not provide recommendations for different classes of obesity (I, II, and III), as defined by WHO. Having a pre-pregnancy BMI in the normal range (18.5–24.9 kg.m^−2^), a GWG within the IOM 2009 guidelines, and losing the excessive weight gain during the postpartum period are associated with better short- and long-term health for the mother and the child (47, 70–73). The accurate knowledge of GWG recommendations by pregnant women is associated with appropriate GWG, as is the correct classification of pre-pregnancy BMI (74). However, there is limited evidence that regular weighting, without a concomitant lifestyle intervention, can control GWG. A recent systematic review and meta-analysis including only two RCTs reported no effect of self-weighting or clinician weighting on GWG per week, or excessive GWG, or other pregnancy, birth and infant outcomes (75).

**Table 2:** The United States of America Institute of Medicine Recommendations (2009) for total weight gain during pregnancy, by pre-pregnancy body mass index.

**Pre-pregnancy BMI**	**BMI (kg.m^−2^)**	**Total weight gain range in kg (lb)**
Underweight	<18.5	12.7–18 (28–40)
Normal weight	18.5–24.9	11.3–15.9 (25–35)
Overweight	25–29.9	6.8–11.3 (15–25)
Obesity (classes I, II, III)	>30	5–9 (11–20)

Pregnant women with obesity often lack knowledge about related complications during pregnancy, and communication with healthcare providers is often experienced as stressful, confusing and judgmental (76). Although health care professionals are well positioned to discuss GWG and healthy behaviors during pregnancy, there are many barriers to patient-provider communication such as lack of clinical guidelines, insufficient training, lack of time, concern about the sensitivity of the topic, negative attitudes and the perception that the advice is ineffective (77). A recent American study has shown that only 52% of pregnant women reported provider's advice on weight gain, 63% on physical activity and 56% on nutrition, though health care professionals can influence women's weight related intentions during pregnancy (76). Women who were less educated, had lower income, were non-White, multiparous and reported lower perceived health, were less likely to report physical activity advice.

The aim of the following overview of systematic reviews and meta-analyses was to identify lifestyle interventions that have shown to be effective in controlling GWG, PPWR and thus improve maternal and child outcomes, in order to inform health care professionals and policy makers.

## Materials and methods

### Study design

A review of international systematic reviews and meta-analyses published in all languages between 1st January 2009 (year of publication of the revised IOM guidelines) (69) and 31st January 2018 was used to identify effective lifestyle interventions to control GWG and/or PPWR.

### Participants, interventions, comparators

Systematic reviews or meta-analyses that evaluated dietary, physical activity, well-being or a multi-component interventions in pregnancy or postpartum were included. They selected only randomized controlled trials (RCT), or provided a separate analysis for RCTs. The main outcome measures were GWG, or GWG above the IOM guidelines, PPWG or postpartum weight loss (PPWL). Studies could include healthy women from any BMI class or parity, with a singleton pregnancy. Comparators are standard care or minimal care or no intervention.

### Search strategy

The Medline, Pubmed, Web of Science and the Cochrane Library databases were used with a combination of the following keywords: (“obesity” OR “overweight” OR “weight gain” OR “weight retention” OR “weight loss” OR “weight management” OR “weight control”) AND (“pregnan^*^” OR “gestation” OR “obstetrics” OR “post-partum” OR “postpartum” OR “postnatal” OR “post pregnancy” OR “post childbirth” OR “following pregnancy” OR “following childbirth”) AND (“lifestyle” OR “behavior” OR “exercise” OR “physical activity” OR “fitness” OR “diet^*^” OR “nutrition” OR “food” OR “well-being” OR “mental health” OR ”psychological health”).

Finally, the keywords (“outcome^*^” OR “complication^*^” OR “co-morbidities” OR “gestational diabetes” OR “hypertension” OR “pre-eclampsia” OR preeclampsia” OR “hemorrhage” OR “hemorrhage” OR “prematurity” OR “stillbirth” OR “macrosomia” OR “large for gestational age” OR ”small for gestational age” OR “dystocia” OR “congenital defect^*^” OR “neonatal complication^*^”) were combined to the primary search to assess effects of interventions on maternal or fetal/neonatal outcomes.

The search was performed by one person (NFL) in the context of a Master's project in Global Health Policy at the London School of Hygiene and Tropical Medicine, University of London. The specificity of the search was increased using search filters for systematic reviews or meta-analyses (Pubmed). The references obtained in the articles were scanned to ensure a complete collection of the relevant systematic reviews and meta-analyses, however no additional articles were found.

### Data extraction

The following data were extracted from the selected systematic reviews and meta-analyses: “a priori” design, search strategy and data, inclusion criteria for selected studies, included studies, countries, participant's characteristics, recruitment, type of interventions, methods of delivery, comparator, outcome measures, quality assessment, analysis, methods used to combine findings, weighted mean difference or risk ratio (and 95% confidence interval), reported quality of evidence, conclusions, source of support and conflicts of interest (for either the review and included primary study authors).

### Quality assessment

Reviews had to report an objective assessment of the methodological quality of studies to assess the risk of bias, as well as heterogeneity and sensitivity analysis (Guidelines of the US National Heart, Lung and Blood Institute) (78). The quality of the selected studies was examined separately by two investigators (NFL and LJE) using the R-AMSTAR checklist—Revised Assessment of Multiple Systematic Reviews for grading of clinical relevance (79). This instrument contains 11 questions (each with 3 or 5 items) rated from 1 to 4 (total score range from 11 to 44). When there was disagreement in the assessment, a consensus was reached through discussion.

### Ethical considerations

The Ethics Committee of the London School of Hygiene and Tropical Medicine of the University of London considered that this research did not require approval.

## Results

### Study selection and characteristics

**Figure 1:**
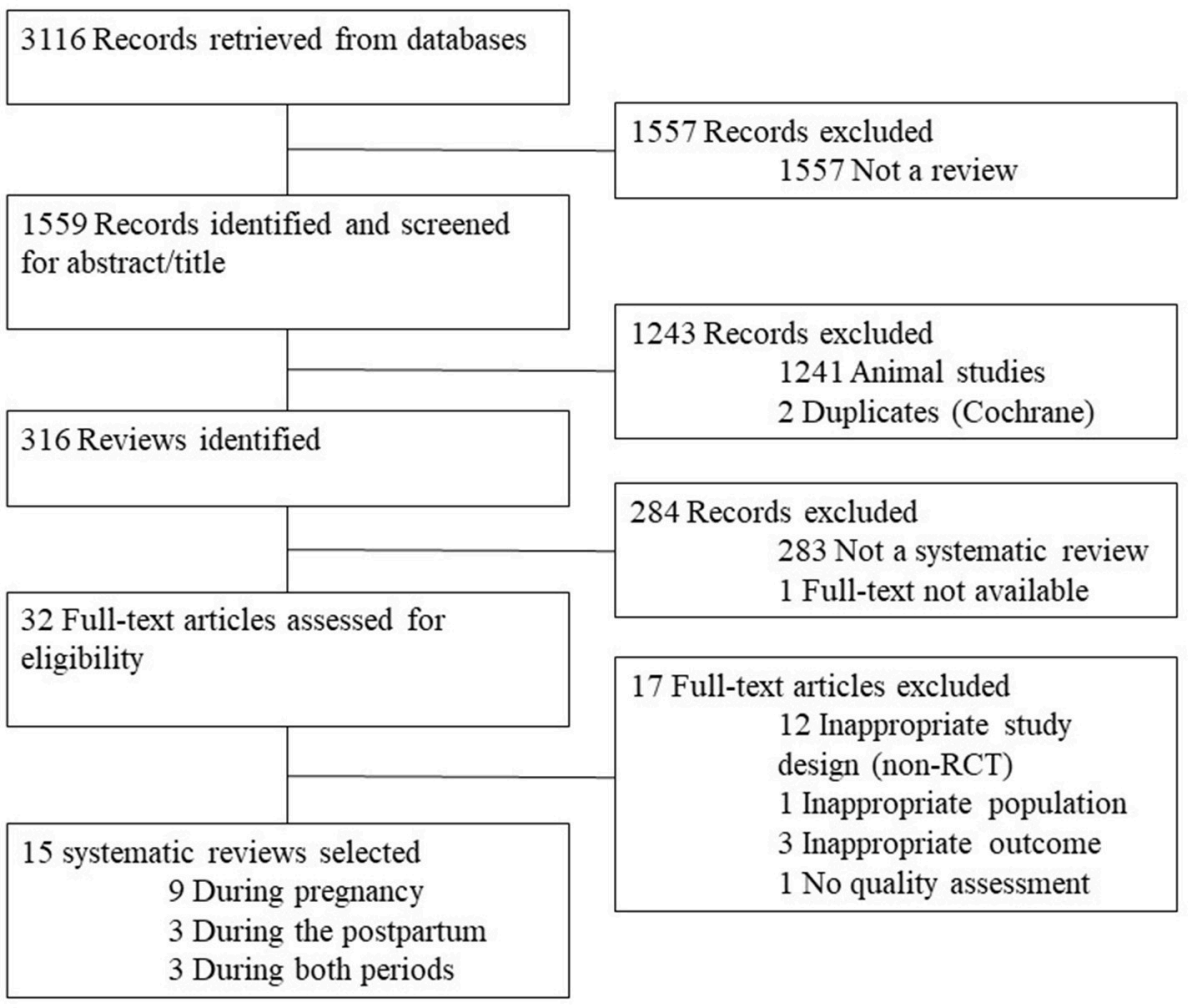
Flow diagram of the systematic reviews retrieved for the review.

The search from the standardized computer databases yielded 3,116 articles (Figure 1). Publications which were not a review were excluded and 1,559 studies were extracted. Titles and abstract were reviewed to identify relevant articles. After removing 1,241 animal studies and 2 duplicates, 316 reviews were identified, of these 32 systematic reviews and/or meta-analyses. The full text for each article was obtained and assessed against the inclusion criteria. Seventeen systematic reviews were excluded due to: inappropriate study design (*n* = 12) (80–91); inappropriate population (*n* = 1) (92); inappropriate outcome (*n* = 3) (93–95) or absence of quality assessment (*n* = 1) (96). Data from the remaining 15 systematic reviews and/or meta-analyses were analyzed to identify effective interventions to control GWG and PPWR/PPWL, and any related impact on maternal and infant outcomes, and contributors to success.

Twelve antenatal and six postpartum reviews of lifestyle interventions on GWG or PPWR were identified (Table 3). Nine reviews also examined the effects of interventions on maternal or fetal/neonatal outcomes (Table 4). The majority of trials were conducted in upper-middle and high-income countries (Australia, Austria, Belgium, Brazil, Canada, China, Colombia, Denmark, Egypt, Finland, Germany, Iran, Italy, Japan, Kosovo, Norway, Sweden, the Netherlands, Spain, Taiwan, Thailand, U.S.A, U.K.). Two studies recruited women with low income in the U.S.A. and Canada. Three reviews conducted a subgroup analysis of antenatal diet, physical activity or multi-component interventions (101, 102, 106); one study examined antenatal and postpartum physical activity interventions (98); and two studies reviewed antenatal and postpartum multi-component interventions (105, 106).

**Table 3:** Summary of effects of lifestyle interventions on gestational weight gain and postpartum weight loss.

**Systematic reviews**	**RCTs (n)**	**BMI**	**Participants (n)**	**R-AMSTAR score**	**% score**	**R-AMSTAR Ranking**	**Weighted mean difference**	**95% CI**	***I*^2^**
**Physical activity interventions during pregnancy**							**GWG**		
Streuling et al. (97)	12	Any BMI	906	33	75	C	−0.61	−1.17 to −0.06	25
Elliot-Sale et al. (98)	3	Any BMI	214	28	64	D	−2.22	−3.14 to −1.30	0
da Silva et al. (99)	18	Any BMI	3,203	30	68	D	−1.1	−1.53 to −0.69	0
Perales et al. (100)	29	Any BMI	Not reported	16	36	D	n.a.		
**Multi-component diet and physical activity interventions during pregnancy**								**GWG**	
Muktabhant et al. (101)	3	Any BMI	444	40	91	A	−1.8	−3.36 to −0.24	76
Thangaratinam et al. (102)	30	Any BMI	3,140^§^	41	93	A	−1.40	−2.09 to −0.71	80
Shepherd et al. (97)	16	Any BMI	5,052	42	95	A	−0.89	−1.39 to −0.40	43
International Weight Management in Pregnancy Collaborative (103)	33	Any BMI	11,410	33	75	C	−0.7	−0.92 to −0.48	0
O'Brien et al. (104)	4	Any BMI	446	30	68	D	−1.25	−2.39 to 0.11	42
Lau et al. (105)	7	OW/OB	1,652	36	82	B	−0.63	−1.07 to −0.20	14
Choi et al. (106)	7	OW/OB	721	30	68	D	−0.91	−1.76 to −0.06	8
Flynn et al. (107)	13	OW/OB	4,276	27	61	D	NA		
**Physical activity interventions during postpartum**							**PPWL**		
Elliot-Sale et al. (98)	2	Any BMI	214	28	64	D	−1.74 (*p* = 0.06)	−3.59 to 0.10	0
**Multi-component diet and physical activity interventions during postpartum**									
Berger et al. (108)	13	Any BMI	1,310	32	73	C	n.a.		
Nascimento et al. (109)	11	Any BMI	769	33	75	C	−2.57	−3.66 to−1.47	66
Lim et al. (110)	32	Any BMI	1,892	29	66	D	−2.3	−3.22 to −1.39	84
Lau et al. (105)^‡^	3	OW/OB	251	36	82	B	−3.6	−6.59 to −0.62	84
Choi et al. (106)	4	OW/OB	547	30	68	D	−1.22	−1.89 to −0.56	25

*Results are presented as weighted mean difference and 95% confidence intervals. RCT, randomized controlled trial; BMI, body mass index; OW, overweight; OB, obesity; GWG, gestational weight gain; PPWL, postpartum weight loss; n.s., non-significant; n.a., not applicable (no meta-analysis)*.

‡ *significant effect at 1-2 months postpartum only*;

§ * Studies on women with pre-existing diabetes or GDM were excluded for this sub-analysis (5 RCTs were excluded)*.

**Table 4:** Summary of effects of lifestyle interventions during pregnancy on relative risks of maternal and neonatal outcomes.

**Systematic reviews**	**RCT (*n*)**	**BMI**	**Participants (*n*)**	**R-AMSTAR Ranking**	**GDM**	**Pregnancy-induced HTA**	**Pre-eclampsia**	**Caesarian section**	**Preterm delivery**	**LGA**	**Macrosomia**	**Neonatal RDS**
**PHYSICAL ACTIVITY INTERVENTIONS DURING PREGNANCY**												
Streuling et al. (97)	12	Any BMI	906	C								
Elliot-Sale et al. (98)	3	Any BMI	214	D								
da Silva et al. (99)	18	Any BMI	3,203	D	0.67 (0.49–0.92)					0.51 (0.30–0.87)		
Perales et al. (100)	57	Any BMI	Not reported	D	Reduced risk (4/14 RCTs, aerobic + resistance training), weak.	Reduced risk (1/12 RCTs, aerobic + resistance training), weak.		Reduced risk (3/15 RCTs, aerobic + resistance training), weak.			Reduced risk (3/21 RCTs, aerobic + resistance training), weak.	
**MULTI-COMPONENT DIET AND PHYSICAL ACTIVITY INTERVENTIONS DURING PREGNANCY**												
Muktabhant et al. (101)	3	Any BMI	444	A		0.70 (0.51–0.96), 5,162 women, low-quality.		0.89 (0.80–1.0, *p* = 0.05), 7,534 women, moderate-quality.			**In OW/OB:** 0.85 (0.73–1.0), moderate-quality	**In OW/OB:** 0.47 (0.26–0.85), moderate-quality.
Thangaratinam et al. (102)	30	Any BMI	Subroup analysis^§^, number not reported.	A	Diet^*^ only: In OW/OB: 0.39 (0.23–0.69), low-quality.	Diet^*^ only: 0.30 (0.10–0.88), low-quality; **In OW/OB**, 0.30 (0.10–0.88), low-quality.	Diet^*^ only: 0.82 (0.43–1.42, NS)		Diet^*^ only: 0.26 (0.09–0.74), low-quality.			
Shepherd et al. (111)	16	Any BMI	6,633	A	0.85 (0.71–1.01), 3633 women, (*p* = 0.07); moderate-quality.			0.95 (0.88–1.02), 6,089 women, moderate-quality.			0.89 (0.78–1.01, *p* = 0.06)	0.56 (0.33–0)
International Weight Management in Pregnancy Collaborative (103)	33	Any BMI	11,410	C				0.91 (0.83–0.99), high-quality.				
O'Brien et al. (104)	4	Any BMI	446	D		0.34 (0.13–0.91)						
Lau et al. (105)	7	OW/OB	1,652	B								
Choi et al. (106)	7	OW/OB	721	D								
Flynn et al. (107)	13	OW/OB	4276	D								

Results are presented as risk ratios, 95% confidence intervals (in brackets) and the reported quality of evidence by authors. BMI, body mass index; OW, overweight; OB, obesity; GDM, gestational diabetes mellitus; HTA, hypertension; LGA, large for gestational age; RDS, respiratory distress syndrome; n.s., not significant. ^‡^significant effect at 1–2 months postpartum only;

§ *Studies on women with pre-existing diabetes or GDM were excluded for this sub-analysis (5 RCTs were excluded), the remaining number of participants was not reported by authors*;

* *Diet only interventions included of a balanced diet consisting of proteins (15–20%), fat (max. 30%) and carbohydrates (50–55%) including low glycemic load (beans, lentils and vegetables, fruits, unprocessed whole grains). ^‡^Significant effect at 1-2 months postpartum only*.

### Participants

Participants were healthy women with a singleton pregnancy or postpartum. Both nulliparous and multiparous women were included. Most antenatal studies recruited participants at less than 20 weeks' gestation. The 15 reviews comprised of five to 65 RCTs, and involved 251 to 11,410 women. Three reviews in pregnancy (105–107) and two reviews in postpartum (105, 106) recruited only women with overweight or obesity. The remaining reviews included women from the general population irrespective of weight status, and the proportion of women with a normal BMI varied widely across trials. Only two reviews reported results for women with overweight/obesity, and those with low-risk (normal BMI) separately (101, 102). One review included women with diabetes, but conducted a subgroup analysis after excluding women with pre-existing diabetes or GDM (102). When ethnicity was examined, most participants were Caucasian or there was insufficient information provided to assess ethnicity.

### Interventions

Twelve reviews examined the effectiveness of interventions that aimed to change lifestyle (diet, physical activity or both) in pregnant women with any BMI (*n* = 9) or with overweight/obesity (*n* = 3). Antenatal dietary interventions typically included a balanced diet consisting of proteins (15–20%), fat (maximum 30%), and carbohydrates (50–55%) with low glycemic load (high fiber: beans, lentils and vegetables, fruits, unprocessed whole grains). Two RCTs provided energy targets by weight (18–24 kcal/kg).

Antenatal physical activity interventions generally consisted of 20–70 min of exercise per day at light to moderate intensity, 2–5 days per week. Selected trials included supervised (*n* = 25, 35–60 min of aerobic and/or resistance training, weight-bearing exercises) or unsupervised (*n* = 8, counseling) physical activity. Pedometers were used in some studies. The multi-component approach included counseling or exercise sessions, education and feedback on weight gain using behavioral change techniques.

Six reviews examined the effectiveness of interventions that aimed to improve lifestyle (diet, physical activity or both) in postpartum women with any BMI (*n* = 4) or with overweight or obesity (*n* = 2). Post-partum interventions were conducted in community, primary care or secondary care settings. Two physical activity only trials included supervised exercise: 45 min of aerobic activity (brisk walking) at 60–70% of maximal heart rate 4 days per week, or walking 10,000 steps per day, during 12 weeks (98). The multi-component diet and physical activity interventions during postpartum comprised of a balanced diet, or a calorie restricted diet, plus supervised (2 trials, walking 3–5 days per week, or general aerobic exercises 5 days per week, or strength training 3 days per week plus walking 10,000 steps per day, during 10–16 weeks) or unsupervised (personalized counseling and skill training, heart rate monitor or pedometer, self-monitoring, feed-back, correspondence programs, text messages, phone calls, Internet) (106, 109, 110). Delivery varied between individual or group sessions, conducted either at home or at a center. The duration of the interventions was 11 days to 36 months.

The comparator in each of the 12 reviews in pregnancy was “usual or standard care.” In the six postpartum reviews, “usual or minimal care,” true control (no intervention) or an alternative concomitant intervention (information printouts) were used as comparators.

### Outcome measures

Eleven reviews in pregnancy examined the same primary outcome measures (GWG, excessive GWG according to the IOM recommendations); one review selected GDM as the primary outcome measure, but included GWG as a secondary outcome (111). Nine reviews in pregnancy examined maternal or fetal/neonatal outcomes as secondary measures (GDM, Pregnancy-induced hypertension, pre-eclampsia, preterm birth, cesarean delivery, birthweight, macrosomia, LGA, stillbirth, shoulder dystocia, neonatal hypoglycemia, neonatal respiratory distress syndrome, admission to intensive care unit). Six of them assessed adverse events (low GWG, SGA, preterm birth, death).

The six postpartum reviews examined PPWR or PPWL as the primary outcome. Two studies assessed maternal outcomes: cardio-metabolic risks (108), or change in moderate to vigorous physical activity and dietary intake (105). Adverse effects were examined in only one of the six postpartum reviews (108).

### Methodological quality of included reviews

The R-AMSTAR assessment results for each review are shown in Table 3. Antenatal reviews scored between 16 (very low-quality) and 42 (high-quality) and postpartum reviews between 29 (very-low quality) and 36 (high-quality), out of a possible 44. Areas where the majority of reviews were marked down included not adequately describing excluded studies and statistical tests, and not providing a clinical consensus statement. However, all reviews reported using the Cochrane Collaboration Risk of Bias tool or the GRADE method.

### Risk of bias

The bias associated with the included trials varied widely across the reviews. Random sequence generation was at low risk of bias for the majority of included studies. Allocation concealment was generally of low or unclear risk of bias. Performance bias was high risk for the majority of the trials, mostly due to the difficulty of blinding study personnel and participants in lifestyle interventions. Detection bias also varied across the reviews, with lower risk of bias for objective outcomes (e.g., body weight). There was an unclear or high risk of attrition bias especially in postpartum trials (drop-out up to 50%). Selective reporting bias was generally unclear or high risk in a large proportion of trials in each review. The proportion of trials with low risk of other biases varied across the reviews.

### Quality of evidence

Four reviews assessed the overall quality of the evidence using the GRADE method (101, 102, 108, 111). Overall the quality of the evidence was high to very low for GWG and moderate to very low for PPWR, and was low for maternal and fetal/neonatal outcomes measured in the reviews and adverse events. There was no data on socioeconomic effects. The reasons for downgrading the evidence included high risk of bias (e.g., attrition), imprecision (wide confidence intervals), and inconsistency (heterogeneity).

### Synthesized findings

#### Lifestyle interventions during pregnancy

##### Physical activity interventions

The summary of effects of antenatal and postpartum lifestyle interventions on GWG (*n* = 12) or PPWR (*n* = 6) is presented in Table 3 (detailed description of reviews in Tables 5–7). Two studies conducted subgroups analysis to assess the effects of physical activity (supervised or unsupervised) interventions on GWG and maternal or child health outcomes (102, 106).

**Table 5:** Systematic reviews and meta-analysis that assessed the effect physical activity interventions in pregnancy.

Quality of reviews^*^	**Systematic reviews**	**Studies included**	**Participants (*n*)**	**Types of intervention**	**Weighted mean difference or summary risk ratio (95% CI)/findings**	**Other findings/Contributors**
**PHYSICAL ACTIVITY INTERVENTIONS**						
**C** (33/44, 75%)	Streuling 2011 (SR+MA) (97)	12 RCTs on GWG.	Any BMI	Light-moderate intensity supervised PA; average frequency 3 days/week; 20–60 min.; aerobic and/or resistance exercises. Duration: from 1st-2nd to 3rd trimester.	Significant reduction of GWG (−0.61 kg; −1.17 to −0.06; *I*^2^ 25%; 906 participants; 12 RCTs).	No dose-dependent effect.
**D** (30/44, 68%)	da Silva 2017 (SR+MA) (99)	18 RCTs on maternal/ infant outcomes (51 cohort studies excluded)	Any BMI	Moderate intensity supervised PA; average frequency 3 days/week; 20–70 min.; aerobic and/or resistance exercises. Duration: from 1st-2nd to 3rd trimester.	Significant reduction of GWG (−1.11 kg; −1.53 to −0.69; *I*^2^ 0%; 3,203 participants; 18 RCTs).	Reduced RR of GDM (0.67, 0.49–0.92; *I*^2^ 33%; 3,790 participants; 10 RCTs) and LGA (0.51, 95% CI 0.30–0.87; *I*^2^ 0%; 1,499 participants; 4 RCTs). No effect on preeclampsia or preterm birth. Difficulties in attending regularly scheduled programs sessions.
**D** (16/44, 36%)	Perales 2016 (SR) (100)	57 RCTs on maternal health or perinatal outcomes	Any BMI	15 trials aerobic exercises; 4, resistance exercises; 30 combined; 8 counseling. 49 RCTs included supervised PA; 23 of them examined effects of supervised PA on GWG. Duration 12–18 week.	Weak evidence for reduced GWG or for higher likelihood of GWG within IOM guidelines after aerobic or aerobic + resistance exercises or counseling.	Combined aerobic and resistance training: strong evidence for improved cardiorespiratory fitness and reduced urinary incontinence. Weak evidence for reduced GDM, pregnancy-induced HTA, duration of labor or cesarean section, and macrosomia after intervention. No adverse outcome.
**D** (28/44, 64%)	Elliott-Sale 2015 (SR+MA) (98)	3 RCTs on GWG, from 1990 only	Any BMI	Light-moderate intensity supervised PA; combined aerobic and resistance exercises; frequency 3–5 days/week; 45–60 min. Duration: 12–33 week.	Significant reduction of GWG (−2.22 kg; −3.14 to −1.3, *I*^2^ 0%; 214 participants; 3 RCTs).	Methodological quality varied considerably across trials. Small number of RCTs.

*BMI, Body mass index; CI, confidence interval; GWG, gestational weight gain; GDM, gestational diabetes mellitus; HIC, high income countries; IOM, Institute of Medicine; LGA, large for gestational age; LMIC, low and middle income countries; MA, meta-analysis; OW, overweight; OB, obesity; PA, physical activity; PPWR, postpartum weight retention; SGA, small for gestational age; RCT, randomized controlled trial; RR, risk ratio; SR, systematic review; wk, week*.

* *The quality of systematic reviews and meta-analysis was assessed using the R-AMSTAR Checklist (ranking, score). When available, the information on the quality of evidence that was reported by authors is indicated in the findings' columns*.

**Table 6:** Systematic reviews and meta-analysis that assessed the effect of multi-component interventions in pregnancy.

Quality of reviews^*^	**Systematic reviews**	**Studies included**	**Participants (n)**	**Type of intervention**	**Weighted mean difference (95% CI)/Findings**	**Other findings/Contributors**
**A** (40/44, 91%)	Muktabhant 2015 (Cochrane SR+MA) (101)	65 RCTs on GWG	All BMI	Dietary counseling (healthy diet or low-fat or low glycemic load or low-energy diet), supervised or unsupervised exercise, or diet and exercise combined. Duration: from the 1st-2nd to the 3rd trimester.	Reduced risk for excessive GWG (RR 0.80, 0.73–0.87; *I*2 52%; 7,096 women; 24 RCTs; high-quality evidence) with PA or combined diet and PA. Five studies reported reduced GWG > 5 kg in intervention vs. control groups. In women with OW/OB, or at risk of diabetes, receiving combined diet and PA interventions, significant reduction of GWG (−0.71 kg, −1.34 to −0.08 kg, *I*^2^ = 57%; 2741 women; 11 RCTs, moderate-quality evidence). Interventions involving low glycemic load, supervised or unsupervised PA, or diet and PA combined all led to similar reductions. Increased likelihood to experience low GWG than those in control groups (RR 1.14, 1.02–1.27; *I*2 3%; 4422 women; 11 RCTs; moderate-quality evidence). Largest reduction accounted with supervised diet and PA.	Reduced RR of gestational HTA (0.70, 0.51–0.96; *I*2 = 43%; 5,162 women; 11 RCTs; low-quality evidence) and macrosomia (with PA interventions, 0.87, 0.71–1.07, *I*^2^ 0%, 2,674 women, 9 RCTs, *p* = 0.05) In women with OW/OB, or at risk of diabetes, reduced RR for macrosomia (0.85, 0.73–1.0; *I*2 0%; 3,252 women; 9 RCTs; moderate-quality evidence) and neonatal respiratory distress syndrome (0.47, 95% CI 0.26–0.85; *I*2 0%; 2,256 women; 2 RCTs; moderate-quality evidence) after combined diet and PA interventions. No effect on preterm birth, pre-eclampsia, LGA or SGA, or other neonatal outcomes.
**A** (41/44, 93%)	Thangaratinam 2012 (SR+MA) (102)	34 RCTs on GWG	All BMI (11/34 trials included OW/OB women)	Balanced diet: proteins (15–20%), fat (max. 30%), carbohydrates (50–55%) with low glycemic index; light to moderate intensity PA (resistance training, weight-bearing exercises, walking) or multi-component interventions (using behavioral change techniques and feed-back on weight gain).	Subgroup analysis (excluding women with pre-existing diabetes or GDM; 30 RCTs): Overall reduction of GWG (−1.4 kg, 95% CI −2.09 to −0.71; *p* < 0.001; moderate-quality evidence). Greater effect with diet only interventions (−5.53 kg, −8.54 to −2.53; *I*^2^ 41%; 6 RCTs, *p* < 0.001), followed by multi-component approach (−1.06 kg, −1.67 to 0.46; *p* < 0.001) and physical activity (−0.72 kg, −1.2 to −0.25, 14 RCTs, P = 0.003). In OW/OB pregnant women, overall reduction of GWG (−2.1 kg, −3.46 to −0.75; *p* < 0.002; *I*^2^ 88%). Diet only interventions, reduction of GWG (−7.73 kg; −6.05 to −9.40; *p* < 0.001; *I*^2^ 41%).	Diet only interventions: significant decrease of risk of gestational HTA (RR 0.30, 95% CI 0.10–0.88) and preterm delivery (0.26, 0.09–0.74). Low-quality evidence. There was a trend toward a reduction of RR of pre-eclampsia (0.82, 0.43–1.42, not significant). In OW/OB women, dietary interventions significantly decreased the risk of gestational HTA (RR 0.30, 0.10–0.88). No other effect on maternal or fetal outcomes (no adverse event, no increased risk of SGA, no effect on birth weight).
**A** (42/44, 95%)	Shepherd 2017 (Cochrane SR+MA) (111)	23 RCTs for preventing GDM	All BMI	Combined diet and PA interventions.	Significant reduction of GWG (−0.89 kg, 95% CI −1.39 to −0.40 kg; Tau2 = 0.37; *I*^2^ = 43 %; women = 5,052; RCT = 16).	Reduced risks of cesarean section (0.95, 95% CI 0.88–1.02; 6,089 women; 14 RCTs; moderate-quality evidence) and respiratory distress syndrome (0.56, 0.33–0; 2,411 women, 2 RCTs). Trend toward a reduction of the risk of GDM (RR 0.85; 0.71–1.01; 6,633 women; 19 RCTs; Tau2 = 0.05; *I*2 42%; *p* = 0.07; moderate-quality evidence) and macrosomia (0.89, 0.78–1.01; 5,368 women, 9 RCTs, *p* = 0.06) in the diet and PA intervention group compared with the standard care group. No difference for pre-eclampsia, pregnancy-induced HTA and/or HTA, perinatal mortality or LGA. No data were reported for infant mortality or morbidity composite.
**C** (33/44, 75%)	i-WIP 2017 (SR+MA) (103)	36 RCTs on maternal and child outcomes.	All BMI (13/36 trials included OW/OB women)	Diet, physical activity or multi-component interventions.	Significant reduction of GWG (−0.70 kg, −0.92 to −0.48 kg, *I*^2^ = 14%; 9,320 women; 33 RCTs, high- quality evidence).	High-quality evidence that interventions reduced the risk of cesarean section (RR 0.91, 0.83–0.99, *I*^2^ 0%; 11,410 women; 32 RCTs), but not for other maternal or child's outcomes.
**D** (30/44, 68%)	O'Brien 2016 (SR+MA) (104)	12 RCTs on GWG.	All BMI	Written information on diet and physical activity + phone calls or regular weighting; visits with a dietician combined with an exercise program 3–5 days/week in 4 trials. Duration: 14–52 week.	Significant reduction of GWG (−1.25 kg; −2.39 to 0.11; *I*2 42%; 446 women; 4 RCTs) and reduced RR for GWG above IOM guidelines (RR 0.72, 0.60–0.8; *I*2 0%; 714 women; 5 RCTs).	Wide variation in the type of intervention, the number of contacts, and the intensity. Reduced RR for hypertension (RR 0.34, 0.13–0.91, I2 0%; 243 women; 2 RCTs). No effect on GDM, preeclampsia, preterm birth, macrosomia or SGA.
**B** (36/44, 82%)	Lau 2017 (SR+MA) (105)	7 RCTs on GWG.	OW/OB	E-based lifestyle interventions (theoretical or conceptual frameworks). Duration: 4 week to 12 months. 7 trials conducted a follow-up up to 12 months.	Significant reduction of GWG (−0.63 kg; −1.07 to −0.20; *I*2 14%; 1652 women; 7 RCTs).	Interventions incorporating in-person (*z* = 2.02, *p* = 0.04), phone (*z* = 2.07, *p* = 0.04) or a combination of in-person and phone delivery formats (*z* = 2.07, *p* = 0.04) were found to be more effective for reducing the GWG in comparison with solely e-based platforms (*z* = 1.10, *p* = 0.27). No effect on birth weight.
**D** (30/44, 68%)	Choi 2013 (SR+MA) (106)	7 RCTs	OW/OB	5 RCTs included supervised light-moderate PA activity 3 days/week or multi-component supervised physical activity 1x/week + diet counseling. Duration: from the 1st-2nd to the 3rd trimester.	Significant reduction of GWG (−0.91 kg; −1.76 to −0.06; *I*2 8%; 721 women; 7 RCTs) after a PA intervention.	Supervised physical activity plus diet showed a significant greater effect on GWG (−1.17 kg; −2.14 to −0.21; *I*2 0%; 372 women; 2 RCTs). Contributors: personalized prescription of PA; goals setting.
**D** (27/44, 61%)	Flynn 2016 (SR) (107)	13 RCTs on GWG.	OW/OB	Diet only or multi-component diet and PA interventions. National recommendations (energy intake 18–24 kcal/kg in 2 trials); individual feedback and alternative healthy choices. Duration 12–30 weeks.	Multi-component interventions: Significant reduction of GWG in 5 of 10 trials in all women and in one trial including only OB women. Diet only interventions: significant reduction in the 3 trials.	Considerable variation in the methodological design of dietary interventions. No evidenced-based approach for any specific dietary regimen. No effect on maternal or neonatal outcomes (no effect on birth weight).

*BMI, Body mass index; CI, confidence interval; OW, overweight; OB, obesity; GDM, gestational diabetes mellitus; GWG, gestational weight gain; IOM, Institute of Medicine; i-WIP, International Weight Management in Pregnancy Collaborative Group 2017; HTA, hypertension; LGA, large for gestational age; MA, meta-analysis; PA, physical activity; PPWR, postpartum weight retention; QCT, Quasi randomized trial; RCT, randomized controlled trial; RR, risk ratio; SGA, small for gestational age; SR, systematic review*.

* *The quality of systematic reviews was assessed using the R-AMSTAR Checklist (ranking, score, %). When available, information on the quality of evidence reported by authors is indicated in the findings' columns*.

**Table 7:** Systematic reviews and meta-analysis that assessed the effect of multi-component diet and physical activity interventions in postpartum.

Quality of reviews^*^	**Systematic review**	**Studies included**	**Participants**	**Types of intervention**	**Weighted mean difference (95% CI)/Findings**	**Other findings/ Contributors**
**PHYSICAL ACTIVITY INTERVENTION**						
**D** (28/44, 64%)	Elliott-Sale 2015 (SR+MA)^(79)^	2 RCTs	All BMI	Individual walking; frequency 4–7 days/week; 45 min; duration 12 weeks.	No significant effect on PPWL (−1.74 kg; 95% CI −3.59 to 0.10, *I*^2^ 0%; 128 women; 2 RCTs).	
**MULTI-COMPONENT DIET AND PHYSICAL ACTIVITY**						
**B** (36/44, 82%)	Lau 2017 (SR + MA)^(80)^	5 RCTs on PPWL	OW/ OB	E-based lifestyle interventions (diet, physical activity and weight management components; theoretical or conceptual frameworks); behavioral goals, counseling and skill training, self-monitoring, feed-back. Duration 4 weeks to 12 months.	Significant PPWL (−3.60 kg; 95% CI 6.59–0.62; *I*^2^ 84%; 251 women; 3 RCTs) during the 1–2 months postpartum. No significant effect in the 6–12 months postpartum.	Significant increase of MVPA at 6 and 13 weeks, and 12 months postpartum (via subjective measures). Significant reduction of caloric intake at 12–20 weeks and 12 months postpartum using the diet-related software measures. No effect on maternal or neonatal complications.
**C** (32/44, 73%)	Berger 2014 (SR)^(85)^	13 RCTs on PPWR (1 diet, 3 PA, 9 combined)	All BMI	Nutrition, exercise or combined diet and PA interventions. Individual counseling, informational pamphlets, telephone calls, text messages, pedometer. Duration 3 to 9 months.	No effect in the 4 good quality RCTs (combined diet and PA). The 4 fair to good quality RCTs reported greater weight loss (from−4.9 to−0.17 kg) in the combined intervention group vs standard care. No effect of diet of PA alone.	No effect on metabolic risk factors or inflammatory biomarkers. Significant reduction of waist-to-hip ratio in one PA trial.
**C** (33/44, 75%)	Nascimento 2014 (SR+MA)^(82)^	11 RCTs on PPWL	All BMI (8/11 RCTs with OW/OB women).	Supervised (4 trials) or unsupervised PA (7 trials; heart rate monitor or pedometer, personalized counseling, correspondence programs, text messages, phone calls, web). Walking or general aerobic exercises were recommended. Resistance exercises combined with walking in one trial. Healthy diet or calorie restricted diet. Duration: 10 to 52 weeks.	Significant PPWL (−2.57 kg; 95% CI −3.66 to −1.47; *I*^2^ 66%; 769 women; 11 RCTs; 4 high-quality trials).	Contributors: Heart rate monitor or pedometer (−4.09 kg; 95% CI −4.94 to −3.25; *I*^2^ 0%; 238 women; 6 RCTs) and exercise combined with intensive dietary intervention (−4.34 kg; 95% CI −5.15 to −3.52; *I*^2^ 0%; 314 women; 6 RCTs).
**D** (29/44, 66%)	Lim 2015 (SR+MA)^(83)^	46 studies on PPWL (32 RCTs/ 14 observational studies).	All BMI (7/32 RCTs with OW/OB women)	Diet, PA or both. 22 RCTs had only a PA component. In-person participation, self-monitoring, individual or group setting, use of technology, home- or center-based intervention. Duration: 11 days to 36 months.	Significant PPWL (−2.30 kg; 95% CI−3.22 to−1.39, I^2^ 84%; 1892 women; 32 RCTs).	Contributors: Combined diet and PA intervention versus PA only (−2.59 kg; 95% CI −3.54 to −1.64; *I*^2^ 79%; 1,359 women; 17 RCTs); Self-monitoring (−2.59 kg, −3.54 to−1.64; *I*^2^ 85%; 1356 women; 17 RCTs); Duration 6 months or less (−3.11 kg; 95% CI −3.54 to −1.64 vs. −1.01 kg; −2.10 to 0.08, *p* = 0.01).
**D** (30/44. 68%)	Choi 2013 (SR+MA)^(77)^	4 RCTs on PPWL	OW/OB	Individual or group sessions on diet and PA; goals setting, self-monitoring, pedometer, telephone call. Restriction of energy intake in 3 trials. Walking; moderate-vigorous intensity; frequency 4–5 times/weeks 30–45 min.; duration 10 to 13 weeks. Supervised in 1 trial.	Significant PPWL (−1.22 kg; 95% CI −1.89 to −0.56; *I*^2^ 25%; 547 women; 4 RCTs)	Contributors: personalized prescription of PA; goals setting.

*BMI, Body mass index; CI, confidence interval; MVPA, moderate to vigorous physical activity; OW, overweight; OB, obesity; PA, physical activity; PPWR, postpartum weight retention; PPWL, postpartum weight loss; RCT, randomized controlled trial*.

* *The quality of evidence of systematic reviews and meta-analysis was assessed using the R-AMSTAR Checklist (ranking, score, %). When available, the information on the quality of evidence reported by authors is indicated in the findings' columns*.

Physical activity interventions were effective in significantly reducing GWG (mean weighted difference, MD −2.2 to −0.61 kg (97–100, 106). The heterogeneity was low (*I*^2^ = 0–25%) and the overall quality of evidence was from low to very low (Table 3). No dose-dependent effect could be demonstrated (80), and some difficulties in attending regularly scheduled programs sessions were reported (99).

The summary of effects of antenatal interventions on maternal, fetal or neonatal outcomes is presented in Table 4 (detailed description of reviews in Table 6). In women from all BMI classes, physical activity interventions were effective in reducing the risk of GDM (−33%, very low-quality evidence) (99), cesarean section (very low-quality evidence) (100) and LGA (−49%, very low-quality evidence) (99) compared to standard care. After excluding the three RCTs with high risk of bias, Muktabhant et al. showed also that the likelihood of macrosomia was significantly reduced (RR 0.56, 95% CI 0.36–0.88, 1,274 women, 8 RCTs, *I*^2^ = 0%) in intervention compared to control groups. Combined aerobic and resistance training significantly increased cardiorespiratory fitness and reduced urinary incontinence (moderate-quality evidence) (100). No adverse effect of physical activity (SGA, preterm delivery) could be identified in two reviews (99, 100).

There was no information in women with overweight or obesity. Though one high-quality review showed in a subgroup analysis that physical activity interventions were effective in significantly reducing GWG (weighted MD −1.35, 95% CI −1.80 to −0.89) in the mixed risk group (all BMI) but not in the high-risk group (women with overweight/obesity or at risk of GMD) (101).

##### Multi-component diet and physical activity interventions

Eight systematic reviews (nine with meta-analysis) included diet and physical activity interventions either as a single or a multi-component program (Table 3).

In pregnant women from all BMI classes, multi-component diet and physical activity interventions were effective in reducing GWG (weighted MD −1.8 to −0.7 kg, *I*^2^ = 0–80%, high-quality evidence in 3 of 5 reviews) (101–104, 111) and in decreasing the likelihood of excessive GWG (RR 0.72–0.80, moderate-quality evidence) (101, 104). There was also evidence for less PPWR (MD −0.94 kg, 95% CI −1.52 to −0.37; 1,673 women, 6 RCTs) at the latest time reported (from 6 weeks to 12 months postpartum) in the antenatal intervention compared to standard care groups (101). Supervised physical activity, personal counseling, weight monitoring or pre-determined maximal GWG goal, and early intervention contributed to reduced GWG.

In pregnant women with overweight or obesity, a significantly reduced GWG (weighted MD −0.91 to −0.63 kg, *I*^2^ = 8–14%, moderate to very low-quality evidence) was also reported in intervention compared to standard care groups (102, 105–107). One high-quality (A) systematic review conducted a subgroup analysis and demonstrated that antenatal diet only interventions (balanced diet, with low glycemic load) had greater effects in reducing GWG in women from all BMI classes (weighted MD −5.53 kg, 95% CI −8.54 to −2.53, *p* < 0.001) or with overweight/obesity (−7.73 kg, 95% CI −9.40 to −6.05 kg, *p* < 0.001, *I*^2^ 41%), compared to standard care (102).

Multi-component diet and physical activity interventions were effective in decreasing the risk of pregnancy-induced hypertension (−66 to −30%; low to very low-quality evidence) (101, 104), cesarean delivery (−9 to −5%; high to moderate-quality evidence) (101, 102) and neonatal respiratory distress syndrome (RDS, −44%, high-quality evidence) (101) in women from all BMI classes. There was a non-significant trend toward reduced likelihood of GDM (−15%, moderate-quality evidence) (111) and macrosomia (−11%) (111).

In women with overweight or obesity, multi-component diet and physical activity interventions were effective in decreasing the risks of pregnancy-induced hypertension (−70%, low-quality evidence) (101, 102), macrosomia (−15%, moderate-quality evidence) (101), but not LGA, and neonatal RDS (−53%, moderate-quality evidence) (101). There was no other effect or harm to maternal or infant health reported. Supervised physical activity or personalized prescription of physical activity, e-based platform plus in-person counseling or telephone calls, contributed to reduce GWG in women with overweight or obesity (105).

Diet only interventions (balanced diet, with low glycemic load) resulted in significantly greater reductions in the risks of GDM (−61%, low-quality evidence), pregnancy-induced hypertension (−70%, low-quality evidence), and preterm delivery (−74%, low-quality evidence) in women from all BMI classes and in pregnancy-induced hypertension (−70%, low-quality evidence) in women with overweight/obesity (102). There was also a trend toward decreased risk of pre-eclampsia (−18%, low-quality evidence), but no evidence of other maternal or fetal/neonatal effect or harm, especially no evidence for SGA.

#### Lifestyle interventions during postpartum

Six systematic reviews (five with meta-analysis) included physical activity (*n* = 1) or multi-component diet and physical activity interventions (*n* = 5, see Table 3);

##### Physical activity interventions

In women from all BMI classes, one review showed that physical activity interventions (12-week progressive walking protocol in 2 RCTs) resulted in non-significant changes in PPWL (13).

##### Multi-component diet and physical activity interventions

In women from all BMI classes, combined diet (healthy diet or calorie restricted diet) and physical activity intervention were effective in reducing PPWR (weighted MD −2.6 to −2.3 kg, very low-quality evidence) (109, 110). Use of a heart rate monitor or a pedometer or modern technologies (internet, text messages, emails, phone calls), self-monitoring, and duration less than 6 months contributed to the effects of interventions (109).

In women with overweight or obesity, combined diet and physical interventions (e-based or individual/ group sessions) were effective to reduce PPWL (weighted MD −3.6 to −1.22 kg, moderate to very low-quality evidence) (105, 106). Personalized prescription of PA and goals setting contributed to PPWL (106). No other maternal effect or harm were observed but there is little data. Dewey et al. reported no change in milk volume and composition among women enrolled in an exercise-only intervention compared to usual care (112).

None of the systematic reviews examined effects of lifestyle interventions on quality of life or psychological health during pregnancy or postpartum.

## Discussion

### Summary of main findings

Multi-component dietary and lifestyle interventions are effective in decreasing GWG and the likelihood of weight gain above the IOM guidelines in women of all BMI classes, without any reported maternal or fetal/neonatal adverse effect. Regular light to moderate intensity physical activity during pregnancy reduce GWG, however interventions including a balanced diet with a low glycemic load, are associated with the greatest reduction. Multi-component diet and physical activity interventions decrease the risks of pregnancy-induced hypertension, cesarean section and neonatal respiratory distress syndrome. Diet in particular is associated with greater reduction of the risk of GDM, pregnancy-induced hypertension and preterm delivery, compared with any other intervention. There is no evidence for effects on outcomes related to fetal weight, morbidity and mortality.

In women with overweight and obesity, multi-component diet and physical activity interventions are effective in reducing the risks of pregnancy-induced hypertension, macrosomia and neonatal respiratory distress syndrome. In addition, diet only interventions are effective in decreasing the risks of GDM and pregnancy-induced hypertension in this population. After delivery, multi-component diet and physical activity interventions are effective in reducing PPWR in women of all BMI classes, but no other effect on maternal or infant outcomes are reported.

### Effective interventions to reduce gestational weight gain

Pregnancy is a time when women may be motivated to change their health behaviors. A healthy diet and regular physical activity are currently recommended during pregnancy in healthy weight pregnant women (69, 113, 114), and women with a BMI over 35 are encouraged to obtain advice from a dietician (115). Our findings support the current recommendations, even if the dietary regimen or the optimal dose of physical activity has not been determined yet. Several components appear to contribute to the control of GWG, such as early intervention implementation, supervised physical activity, personal counseling, weight monitoring combined with a lifestyle intervention, or pre-determined maximal GWG goal.

An increased energy intake during the 2nd and the 3rd trimester is usually recommended (116). Our evidence review shows that diet-based interventions (counseling, balanced diet, low glycemic load, energy target by weight 18–24 kcal/kg, food diary) are associated with the greatest reduction in GWG. Therefore, caution should be taken in women with overweight and obesity in telling them to increase their energy intake in the 2nd and 3rd trimesters.

The epigenetic profile of the developing fetus is sensitive to environmental influence. Maternal diet has been shown to influence DNA methylation patterns in offspring, but research in humans is limited (117). Recently, findings from the ROLO study (Randomized control trial of Low glycaemic index diet to prevent macrosomia) suggested that low glycemic index dietary intervention during pregnancy was associated with subtle, yet widespread differential DNA methylation at regions across the offspring's genome (118). These data imply that exposure to a dietary intervention may impact the neonatal epigenome and therefore their risk of obesity and NCDs during fetal development, though larger studies are required to fully explore interventions in pregnancy.

Women typically reduce their physical activity level during pregnancy (119). This evidence review shows that light to moderate intensity physical activity, including aerobic and resistance exercises, should be encouraged 3 −5 times per weeks for a duration of 30–60 min without adverse effects in healthy pregnant women. Our findings are in line with the American College of Obstetricians and Gynecologists (ACOG) who recommends that pregnant women should engage in moderate exercise for 30 min per day on most days of the week, with the exception of women with compromising health conditions (e.g., pre-eclampsia) (114). The anatomic and physiological changes, absolute and relative contraindications should be considered. Activities that increase the risk of falls or those that may result in excessive joint stress, should include cautionary advice for most pregnant women, but evaluated on an individual basis with consideration for individual abilities. The major challenge remains how best to engage pregnant women in regular physical activity and sustain changes during the perinatal period. Lau et al. has shown that a combination of in-person, e-based and phone interventions is more effective to reduce GWG, in comparison with an e-based platform alone.(80) It is possible that women with overweight or obesity need a higher dose of physical activity to influence GWG or other pregnancy outcomes compared to normal weight women. Evidence supports moderate intensity physical activity between 150 and 250 min per week to be effective to prevent weight gain in adults with overweight or obesity (120), however there is to date no information in pregnant women.

The effects of antenatal interventions on maternal and fetal morbidities and mortality remained unclear. This review of reviews demonstrates that there is low-quality evidence that multi-component diet and physical activity interventions decrease the likelihood of pregnancy-induced hypertension, cesarean section and neonatal respiratory distress syndrome in women from all BMI classes. Furthermore, diet-based interventions were shown to be effective in decreasing the risks of GDM and pregnancy-induced hypertension in women with overweight and obesity. These findings are of particular importance to primary care providers, as pre-pregnancy obesity is an independent risk factor for serious maternal complications (3, 13–16, 23, 24).

Although the quality of evidence remain low, the available evidence suggests that antenatal multi-component lifestyle interventions are also effective in reducing the risk of macrosomia and neonatal respiratory distress syndrome in women with overweight and obesity (3). As maternal pre-pregnancy obesity is associated with an increased risk for the offspring developing childhood obesity and NCDs in the long term, these findings suggest that health care providers should pay particular attention to this high-risk population, in order to prevent the vicious intergenerational cycle of obesity (3, 33, 40).

Combined diet and physical activity lifestyle interventions are effective to reduce GWG, with evidence from this review showing a decrease by 20–28% in the risk for GWG above the IOM guidelines, although the magnitude of effects on weight is small (−1.8 to −0.7 kg). So weight loss prior pregnancy is probably needed to achieve both GWG goals and optimal pregnancy outcomes (36, 115, 121). The current European Association for the Study of Obesity (EASO) guidelines for the management of adult obesity provide useful information for primary care providers (122). This treatment should be undertaken by a multidisciplinary obesity team with the ability to tackle the different aspects of obesity and its co-morbidities. Weight loss objectives should be realistic (5–10% over a period of 6 months) and individualized. Structured intensive programs using cognitive-behavioral techniques in individual or group setting are effective in achieving realistic goals in an adequate time frame (123).

Whilst bariatric surgery is currently the most cost-effective treatment resulting in substantial weight loss in carefully selected patients, it should only be considered for those patients with severe obesity, or help with co-morbidity management (124). Available data suggests that pregnancy following bariatric surgery is associated with improved maternal and fetal outcomes, compared to women with untreated obesity, however it is also related to premature delivery and increased risk of SGA (125). Pregnancy is therefore not recommended 12–18 months after surgery, and the antenatal care of women how have undergone bariatric surgery should be undertaken at a specialized center (126).

### Effective interventions to reduce postpartum weight retention

The postpartum period is also a window of opportunity to encourage women to lose excessive weight at a time when they are usually motivated. This evidence review shows that interventions that include both diet and physical activity components, and comprise individualized support and self-monitoring are more likely to be successful in reducing PPWR in all BMI categories. However the optimal approach to reduce PPWR remains uncertain. Ostbye et al. observed that home-based interventions provided a less burdensome and more practical approach than clinic-based attendance (127). Interventions delivered via email, mail/post, telephone, text messaging or the Internet appear to be more practical for postpartum women than traditional face-to-face methods. These methods of delivery for weight loss management have also been successful in the general population (128). Web-based weight management programs have also been found to be as successful as traditional face-to-face counseling for short-term weight loss (129). As women from lower socioeconomic backgrounds are at higher risk of developing obesity after birth, they should be targeted for PPWL interventions (130).

The ACOG recommends a gradual return to physical activity 4–6 weeks after Childbirth (131), however, several studies observed that a high proportion of women are not as active as recommendations advise during the year following childbirth (132). This current review demonstrates that physical activity–based interventions have no effect on PPWR (98), compared to multi-component diet and physical activity approaches (105, 106, 108–110). Dewey et al. (112) suggested that in absence of a proper dietary intervention, women tend to increase their caloric intake as their energy expenditure through exercise increases, thus the calorie deficit required for weight loss cannot be reached resulting in an ineffective intervention. Light to moderate physical activity itself may not be sufficient to induce weight loss after birth weight. However, physical activity during the post-partum period can induce other beneficial effects on health, such as increased cardiovascular fitness (133) or reduced depression symptoms after child birth (134).

### Limitations

Our review of systematic reviews and meta-analyses provides a comprehensive and up to date (up to January 2018) overview of the current reviewed evidence. A rigorous quality assessment was undertaken by two independent reviewers for each review (R-AMSTAR). The quality assessed in each systematic review ranged between high to very low for the benefit observed with GWG and PWWR, but low for other important maternal or neonatal outcomes. The low evidence rating was explained by significant heterogeneity observed in the effect size, risk of publication and related biases, and deficiencies in the quality of the study. There were large differences in the types of interventions and participants, mode of delivery, timing of the measurements and implementation of intervention, dose of intervention, and how it was monitored and supervised. Most included studies were also carried out in middle-high and high-income countries and it is not clear whether these findings are applicable to low income settings.

### Implication for research

Future research should focus on: the optimal dose (type, frequency, intensity and duration) as well as the level of supervision in interventions that aim to reduce GWG and PPWR; measurements of psychosocial determinants of GWG and PPWR; interventions in various groups based on BMI, age, ethnicity, socioeconomic status, parity, and risk status in pregnancy; the sustainability and long term effects of the interventions on the mother and child; the cost-effectiveness of the interventions and their feasibility in terms of incorporation into clinical settings; and strategies to improve the adherence and compliance of lifestyle interventions. Trials should be more systematically designed evaluated and reported. The optimal amount of weight gain or loss during pregnancy that would minimize maternal and fetal complications remains a topic of discussion (64–68), and there is a need to examine the tailoring of interventions to the severity of maternal obesity.

## Conclusions

The burden of obesity not only threatens global health care systems but also the potential to cripple national economies and global development (135). The perinatal period seems to be a critical windows of opportunity to influence long-term obesity and NCDs risk for women and their child, as well as maternal weight status for subsequent pregnancies. Adopting healthy behaviors may also contribute to a healthier lifestyle for the whole family and to the prevention of early childhood obesity (136). Evidence-based recommendations and training programs for health care professionals are urgently needed to increase the proportion who discuss these topics, and who do so accurately (131). Despite the above limitations, some clear conclusions can be made concerning the type and dose of interventions.

A multi-component approach including a balanced diet, with low glycemic load, and light to moderate physical activity, 30–60 min per day 3–5 days per week, should be recommended from the first trimester of pregnancy and maintained during the postpartum. As there is no evidence that the interventions evaluated in this review are associated with adverse maternal or fetal outcomes, we conclude that that desirable outcomes of lifestyle interventions outweigh possible harms. Dietary interventions appear to be most effective in reducing GWG and co-morbidities such as gestational hypertension and preterm birth in the general population, but also pregnancy-induced hypertension, GDM and macrosomia in women with obesity. We hope that this evidence review will serve as a basis to inform new policies on maternal and child health to halt the intergenerational cycle of obesity.

## Author contributions

NF-L contributed to the conception and design of the study, conducted the review of reviews, assessed the quality of studies, drafted and revised the manuscript. CS and LE provided input into the conduct of the study, the grading of evidence and the interpretation of data. BM contributed to the review of maternal and fetal complications associated with obesity and excessive GWG and to the interpretation of data. All authors contributed to the manuscript revision, read and approved the submitted version.

### Conflict of interest statement

The authors declare that the research was conducted in the absence of any commercial or financial relationships that could be construed as a potential conflict of interest.

## Abbreviations

BMI: Body Mass Index
CVD: Cardiovascular disease
GDM: Gestational diabetes mellitus
GWG: Gestational weight gain
GWL: Gestational weight loss
HTA: Hypertension
IGR: Intra-uterine growth retardation
IOM: Institute of Medicine
LGA: Large for gestational age
NCD: Non-communicable disease
OB: Obesity
OW: Overweight
PA: Physical activity
PPWL: Post-partum weight loss
PPWR: Post-partum weight retention
PTB: Preterm birth
RCT: Randomized controlled trial
SGA: Small for gestational age
WHO: World Health Organization.
